# Mechanistic insights into the primary and secondary alterations of renal ion and water transport in the distal nephron

**DOI:** 10.1111/joim.13552

**Published:** 2022-08-21

**Authors:** Nahid Tabibzadeh, Gilles Crambert

**Affiliations:** ^1^ Laboratoire de Physiologie Rénale et Tubulopathies Centre de Recherche des Cordeliers INSERM Sorbonne Université Université Paris Cité Paris France; ^2^ EMR 8228 Unité Métabolisme et Physiologie Rénale CNRS Paris France; ^3^ Assistance Publique Hôpitaux de Paris Hôpital Bichât Paris France

**Keywords:** acid and base balance, blood pressure, kidney, potassium, tubulopathies

## Abstract

The kidneys, by equilibrating the outputs to the inputs, are essential for maintaining the constant volume, pH, and electrolyte composition of the internal milieu. Inability to do so, either because of internal kidney dysfunction (primary alteration) or because of some external factors (secondary alteration), leads to pathologies of varying severity, leading to modification of these parameters and affecting the functions of other organs. Alterations of the functions of the collecting duct (CD), the most distal part of the nephron, have been extensively studied and have led to a better diagnosis, better management of the related diseases, and the development of therapeutic tools. Thus, dysfunctions of principal cell–specific transporters such as ENaC or AQP2 or its receptors (mineralocorticoid or vasopressin receptors) caused by mutations or by compounds present in the environment (lithium, antibiotics, etc.) have been demonstrated in a variety of syndromes (Liddle, pseudohypoaldosteronism type‐1, diabetes insipidus, etc.) affecting salt, potassium, and water balance. In parallel, studies on specific transporters (H^+^‐ATPase, anion exchanger 1) in intercalated cells have revealed the mechanisms of related tubulopathies like distal renal distal tubular acidosis or Sjögren syndrome. In this review, we will recapitulate the mechanisms of most of the primary and secondary alteration of the ion transport system of the CD to provide a better understanding of these diseases and highlight how a targeted perturbation may affect many different pathways due to the strong crosstalk and entanglements between the different actors (transporters, cell types).

## Graphical abstract

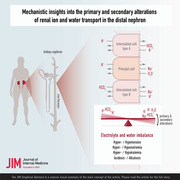

## Introduction

**Fig. 1:**
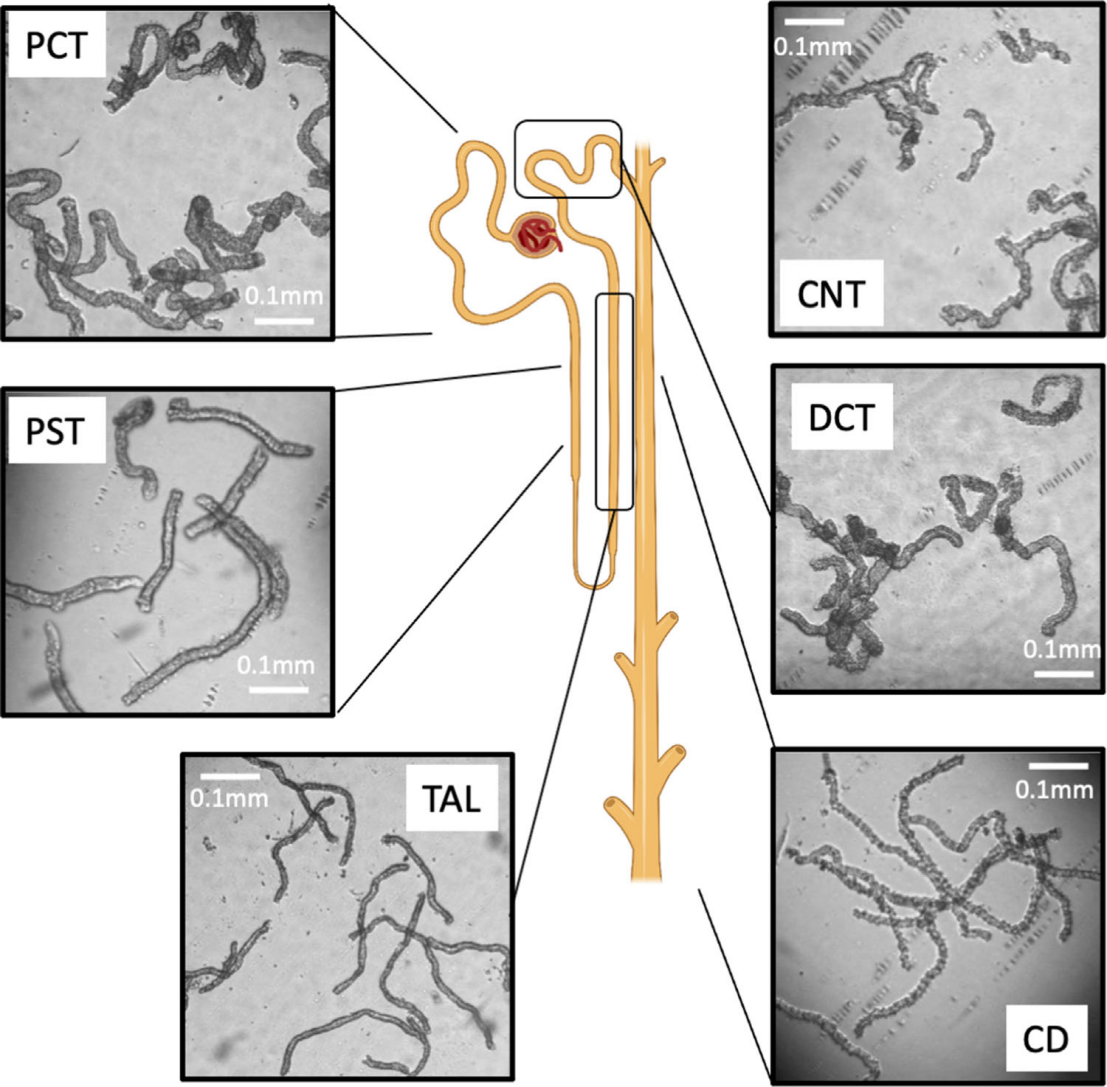
Schematic representation of a nephron with pictures of different segments isolated manually under a binocular loupe. CD, collecting duct; CNT, connecting tubule; DCT, distal convoluted tubule; PCT, proximal convoluted tubule; PST, proximal straight tubule; TAL, thick ascending limb.

Life strongly depends on the ability of an organism to maintain its “milieu intérieur” within narrow limits, which permits it to move into different environments and to sustain strong variability in diets and fluid consumption. The kidney is the key organ for this process, allowing to maintain the hydro‐electrolytic balances of the organism since it equilibrates the outputs with the inputs. This simple function, easy to enunciate, is a sum of multiple mechanisms adjusting very finely and in a timely manner the excretion of ions and water to the ingested amounts. Any disturbance in this global function leads to physiological disorders of varying severity with the ability to impact the functions of all the organs and tissues of the body. The kidney is organized and highly structured around its functional unity, the nephron, and a vascular system. The nephron consists of a glomerulus, which is the filter where tufts of capillaries are embedded into an epithelial structure (Bowman's capsule), and a tubular epithelial structure. This tubule is divided into segments that differ anatomically, are composed of specific cells, and have specific functions (Fig. 1). Moreover, the embryonic origin of these segments is different; the glomerulus and the most proximal parts up to the connecting tubules (CNTs) emerge from metanephric mesenchyme and connect to the collecting duct (CD) originating from the uretheal bud (for review, see [1]).

In this review, after a brief description of the nephronic structures and their functions, we will mainly focus on the physiopathological roles played by the CDs by describing how genetic modifications or environmental interaction interfere with its functions and lead to pathologies.

### Functional specificity of renal segments and associated pathologies

**Fig. 2:**
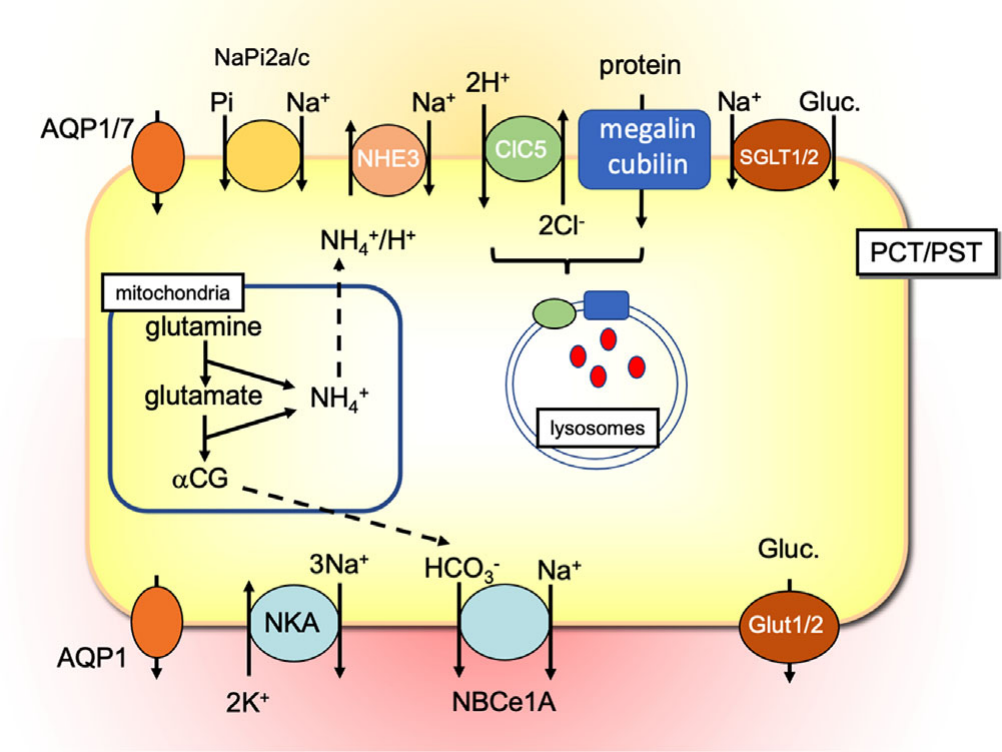
Schematic representation of an epithelial cell of the proximal tubule showing the main ion transporters and some regulators.

*Proximal tubules*. The proximal tubule, subdivided into three segments (proximal convoluted tubule [PCT], proximal straight tubule, and proximal segment 3 [S3]), represents roughly 80% of the renal epithelial cells. It plays multiple roles (Fig. 2), mainly by reabsorbing large amounts of molecules (low molecular mass proteins, amino‐acids, sugars, water) and ions (P_i_, K^+^, HCO_3_^–^, Na^+^, etc.). For instance, it reabsorbs around 85% of the filtered P_i_ and 70% of the filtered Na^+^. Proximal tubules also play a central role in acid–base regulation by reabsorbing the majority of filtered bicarbonate and producing ammonia, allowing protons to be secreted under the ammonium form (for review, see [2]). Because of these general functions, the alteration of the proximal tubule may have global effects, known as the renal Fanconi syndrome and characterized by a loss of Pi, leading to hypophosphatemia, of glucose, amino‐acids, and proteins in addition to the development of renal tubular acidosis and apparition of cystinosis [3]. More specific alterations lead to isolated syndromes; for instance, an X‐linked mutation of the *CLCN5* genes (encoding for a chloride/proton exchanger) causes the Dent syndrome [4]. Pharmacological treatments also impact the proximal tubule functions. For instance, the anticancer drug, cisplatin, specifically targets and kills the proximal tubule cells of the S3 segment, leading to tubular damage and dysfunction with Na^+^, K^+^, and Mg^2+^ wasting that may, ultimately, end in acute kidney disease [5]. This cell specificity is due to the expression of OCT2 (organic cation transporter 2) at the basolateral membrane of the proximal tubule cells, allowing cisplatin to be highly concentrated in their cytoplasm [6]. On the other hand, proximal tubule cells may be purposefully targeted to treat diseases. Thus, a recent major advance in the treatment of diabetes and hypertension concerns the inhibition of a sodium/glucose transporter (SGLT2) localized at the apical side of the proximal tubule cells. This inhibition has demonstrated a remarkable efficiency in the treatment of diabetes by allowing the kidney to excrete glucose [7], but it is also a promising therapy to decrease blood pressure [8] that is or is not associated with diabetes, and has proven efficient in reducing GFR (Glomerular Filtration Rate) decline in proteinuric chronic kidney diseases.

**Fig. 3:**
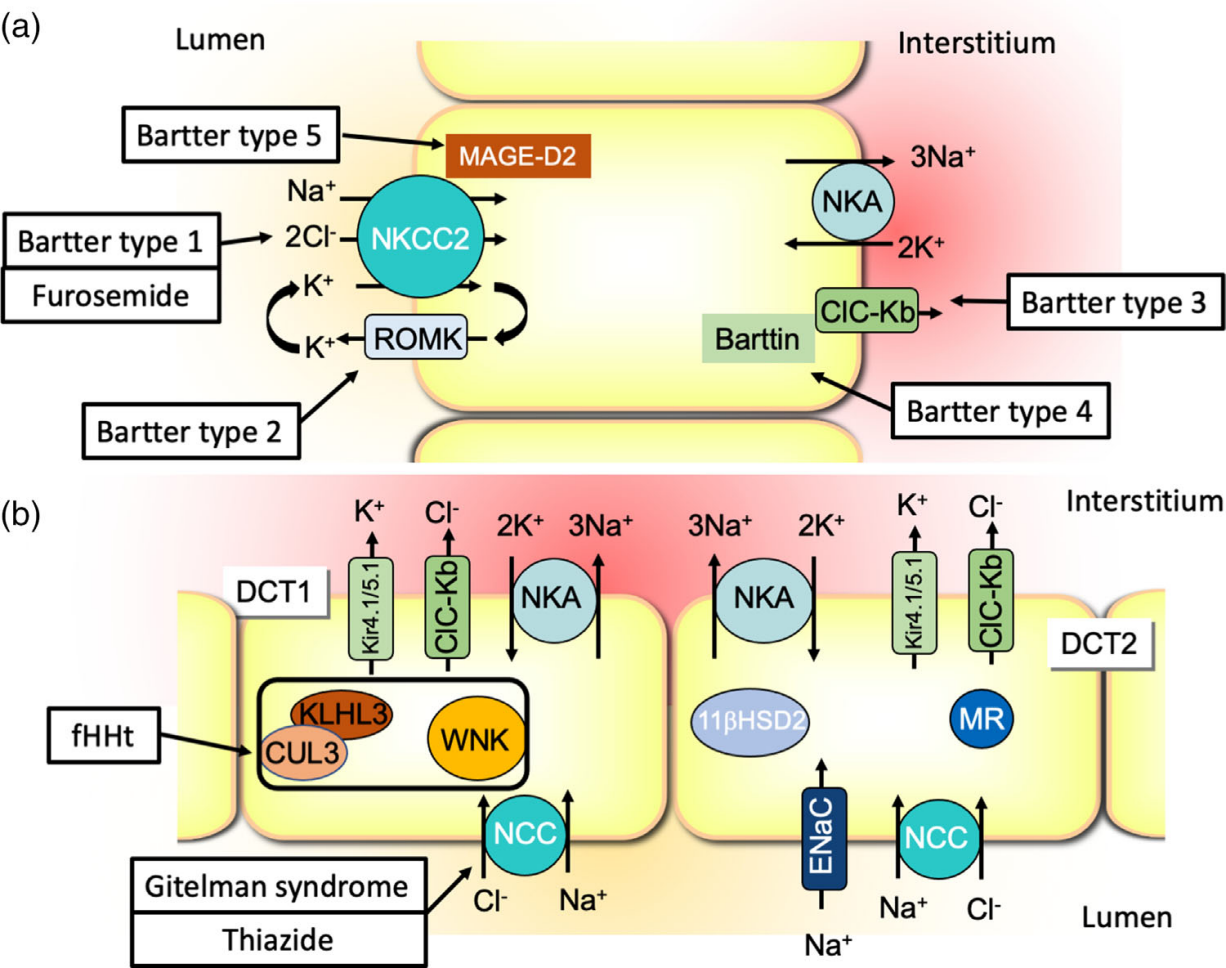
(a) Schematic representation of an epithelial cell of the thick ascending limb showing the main ion and solute transporters with their correlated diseases and the pharmacological treatment targeting this cell type. (b) Schematic representation of an epithelial cell of the distal convoluted cell showing the main ion and solute transporters with their correlated diseases and the pharmacological treatment targeting this cell type.

*The Henle loops*. The proximal tubule is followed by thin descending and thin ascending limbs, which form the tip of the Henle loop in the medulla. The last part of the Henle loop is the medullary and the cortical thick ascending limb. These segments are of particular importance for salt, calcium, and magnesium reabsorption. The impermeability of the TAL to water drives the accumulation of salt in the interstitium that contributes to the generation of the corticomedullary osmotic gradient, required for water conservation. Although it is generally considered as a whole, medullary and cortical parts of the TAL have distinct functions (for a recent review, see [9]). Because of its great ability to reabsorb NaCl (25% of the filtered load) and its importance in water balance, inhibition of TAL function by furosemide (which targets the Na,K,2Cl‐cotransporter 2, NKCC2) is a very efficient therapeutic way to decrease extracellular volume in case of edema and ascites and to reduce blood pressure in hypertensive patients. Genetic disorders (Bartter syndrome types 1–5) involving mutations in specific TAL transporters or regulatory proteins (Fig. 3a) recapitulate the effect of furosemide, leading to massive salt and water wasting and the development of hypokalemia (for review, see [10]).

*The distal convoluted tubules*. The distal convoluted tubule (DCT) is a short segment that is very important for salt and K^+^ balance. The DCT makes the transition between TAL and connecting/collecting tubules and is the site of intense ion transport. It can be split into DCT1 and DCT2, which are composed of different cells and can be distinguished by their response to mineralocorticoid and their equipment in ion transporters (Fig. 3b). Indeed, although the mineralocorticoid receptor (MR) is expressed in all DCT cells, the DCT2 response to aldosterone is specific because of the expression of the 11βHSD2 enzyme, which inactivates glucocorticoids. The Na/Cl cotransporter (NCC) is expressed in all DCT cells but only those of DCT2 also express the amiloride‐sensitive Na^+^ channel (ENaC). In addition to its strong ability to reabsorb Na^+^ and Cl^–^, the DCT has recently emerged as a crucial segment in the balance of K^+^. Indeed, although it does not contribute directly by itself to K^+^ secretion or reabsorption, DCT controls the Na^+^ delivery to the downstream segments that secrete K^+^ in an Na^+^‐dependent manner (see below). For instance, in the case of K^+^ depletion, DCT cells proliferate to enhance the surface of Na^+^ reabsorption [11] and hypokalemia induces NCC activation through a pathway that involves the basolateral K^+^ channel Kir4.1/Kir5.1 and CLC‐Kb [12, 13, 14]. By these processes, along with K^+^ depletion, the Na^+^ is mainly reabsorbed in DCT, which impedes the Na^+^‐dependent K^+^ secretion in CD (see below). Conversely, when K^+^ intake is high, the Na^+^ reabsorption in DCT is blunted to increase the Na^+^ delivery to CD and increase the Na^+^‐dependent K^+^ secretion [15]. The importance of the DCT has been proved by the strong defects generated by many genetic diseases and by the effect of the thiazides that inhibit NCC. Thiazides are one of the most potent antihypertensive drugs, indicating that the salt reabsorption in DCT is highly contributive to the volume of the extracellular compartment. As for genetic diseases, loss‐of‐function mutations of NCC lead to Gitelman syndrome, exhibiting thiazide‐like effects with a loss of salt and the development of hypokalemia. The mirror situation is known as the familial hyperkalemic hypertension disease or Gordon syndrome, or type 2 pseudohyperaldosteronism. Here, NCC is hyperactive due to mutations of some of its regulatory proteins (WNK1, WNK4, CUL3, or KLHL3) inducing strong reabsorption of salt. Therefore, it leads to marked hypertension and hyperkalemia (for a broad review of DCT function and related diseases, see [16, 17]).

*The CNTs and CDs*. Both CNT and CD consist of principal cells (PC), among which are interspersed intercalated cells (IC); the latter is subdivided into type A (or alpha, ICA), type B (or beta, ICB), and non‐A non‐B cells. However, depending on the segments, what are called PC are not completely equivalent (see below). Moreover, in mouse CNT, the majority of the IC are of non‐A non‐B types, with functions that remain to be established, whereas in rats, this type of IC is rare in all segments [18]. Interestingly, CNT emerges in the zone of contact of the two embryonic structures that form the nephron. The CNT literally connects the segment of metanephric mesenchyme origin (from PCT to DCT) to that of ureteral bud (CD). This may be the reason why the cell composition of this segment is similar but not completely identical to those found downstream, in CD. For instance, the PC of the CNT, expressing aquaporin 2, ENaC, and the MR also possess an efficient and specific system for transcellular Ca^2+^ reabsorption. These PC indeed express an apical Ca^2+^ channel, TRPV5, and two basolateral Ca^2+^ transporters, a pump (PMCA1), and an Na/Ca‐exchanger (NCX1). The CNT‐specific Ca^2+^ reabsorption is under the control of the parathyroid hormone and roughly contributes to 15% of the filtered load of Ca^2+^ (for review, see [19]).

**Fig. 4:**
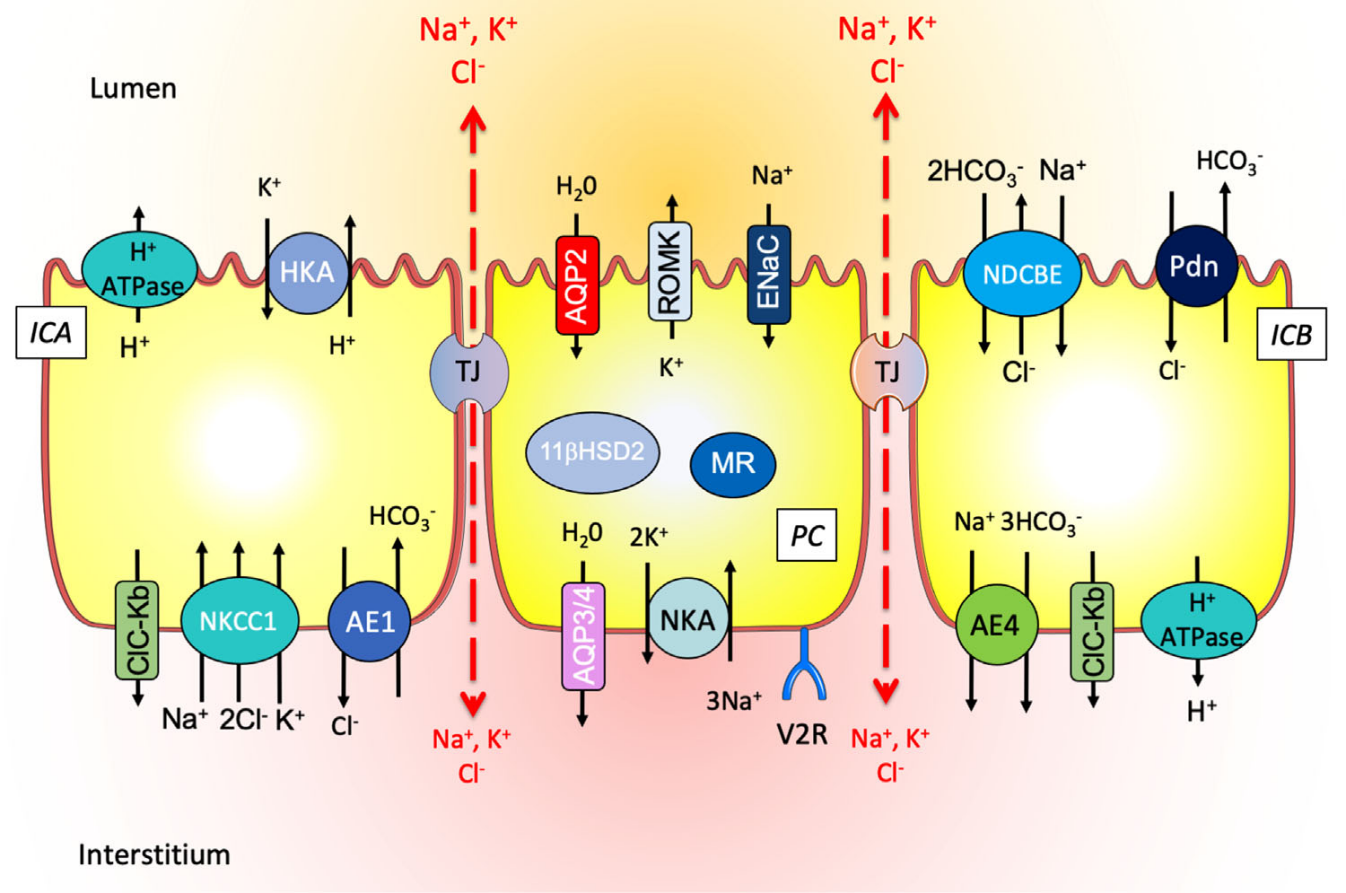
Schematic representation of the epithelial cells of the collecting duct showing the main ion and solute transporters.

Regarding the CD (Fig. 4), although its contribution to ion transport is weak compared to other segments, it is considered a crucial structure for ion and water balances since it is the last part of the nephron where regulations can take place to exactly match the excretion to the intakes. The dogma established years ago clearly ascribed specific functions to the different cell types. Thus, PC mainly contributes to Na^+^ reabsorption, K^+^ secretion, and water reabsorption under the control of two main hormones, aldosterone and vasopressin. They express ENaC, ROMK, and AQP2 channels at their apical side and Na,K‐ATPase and AQP3/4 at the basolateral side. ICA and ICB are involved in acid and bicarbonate secretion, respectively. ICA exhibits an apical H^+^‐ATPase and a basolateral Cl^–^/bicarbonate exchanger (AE1 or band 3), allowing secretion of proton and reabsorption of bicarbonate. They also express the H,K‐ATPase type 2 that is stimulated to reabsorb K^+^, for instance in case of K^+^ depletion or gestation, under a progesterone‐dependent pathway [20, 21]. In ICB, the H^+^‐ATPase is basolateral and a Cl^–^/bicarbonate exchanger, the pendrin (slc26a4), is present at the apical side, allowing secretion of bicarbonate and reabsorption of proton.

This clear distribution of tasks was amended in the early 2000s, when studies started to identify the pendrin, a Cl^–^/bicarbonate exchanger, specifically expressed in ICB, as a major component of NaCl reabsorption, contributing to the identification of an ICB‐specific electroneutral NaCl reabsorption pathway [22, 23]. Later, ICA was also shown to participate in the salt balance through the presence of an Na^+^ secretion pathway involving the basolateral entry of Na^+^ by the activity of Na,K,2Cl‐cotransporter 1 (NKCC1), and its apical exists through H,K‐ATPase type 2 (HKA2) working as an apical Na,K‐ATPase [24]. It therefore turns out that the simple functional classification of CD cell functions is a bit more complicated than generally illustrated, giving rise to a novel paradigm where the different cell types may not have a unique function but instead contribute to the complex system that is required to maintain ionic balance.

Interestingly, the different cell types also “communicate” with each other and mutually interfere with their functions via paracrine systems. As an example, there is a cross‐talk between PC and ICA during acidosis where this situation is sensed by the PC, through a vasopressin‐dependent process, leading to the production of a growth factor, GDF15. This peptide then acts on the ICA to promote their proliferation [25, 26].

**Fig. 5:**
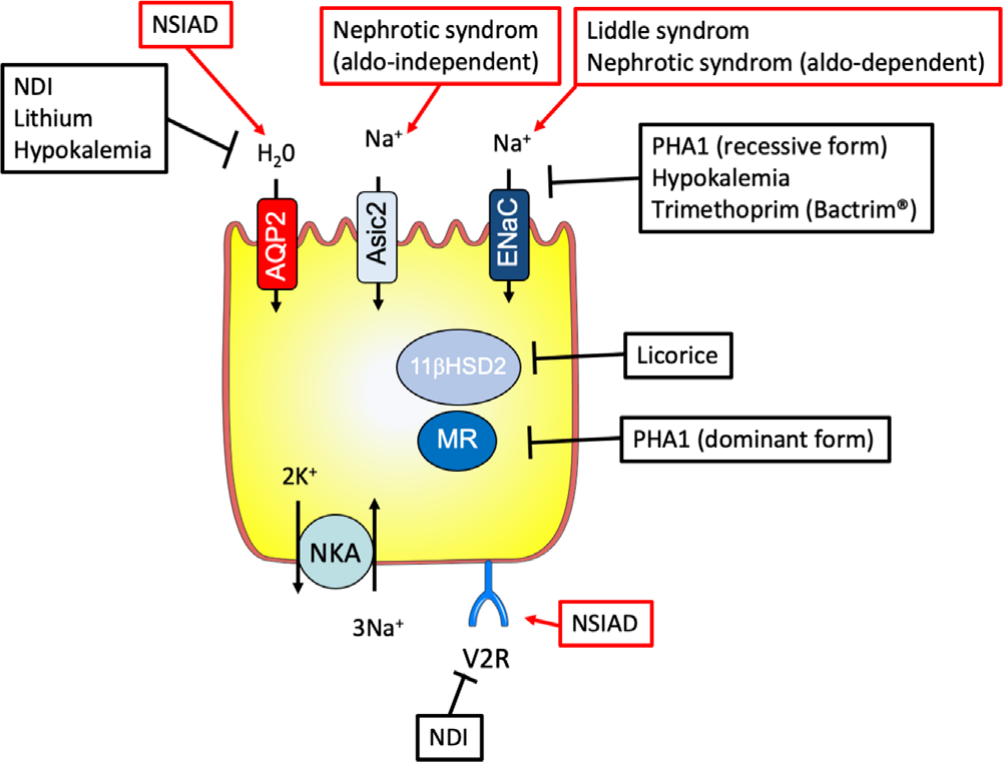
Schematic representation of the principal cell of the collecting duct recapitulating proteins involved in primary (in black) and secondary (in red) perturbations of the collecting duct.

**Fig. 6:**
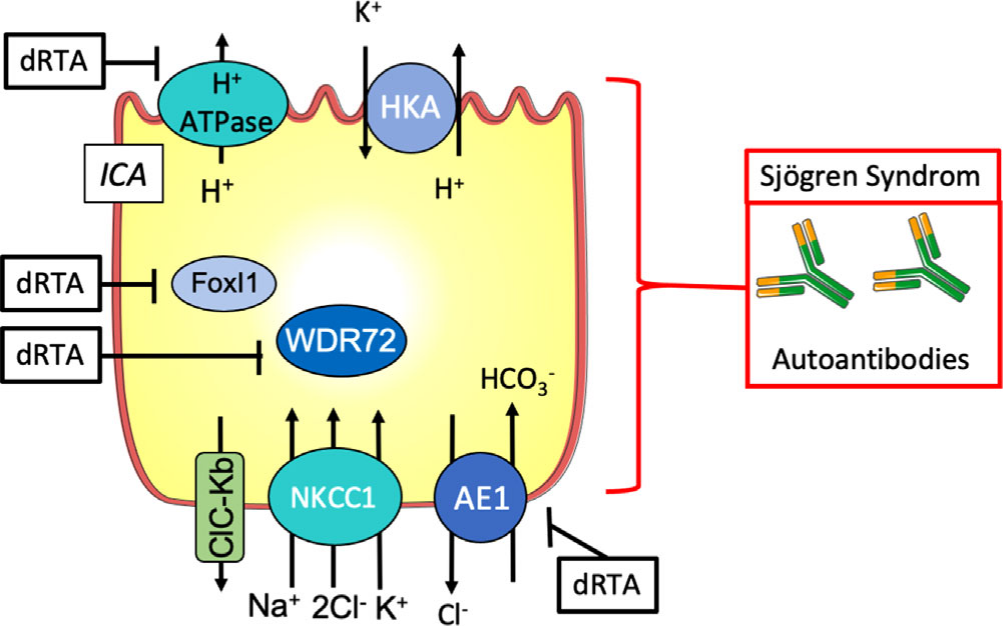
Schematic representation of the type A intercalated cells of the collecting duct recapitulating proteins involved in primary (in black) and secondary (in red) perturbations.

In view of the crucial roles played by CD in the Na^+^, K^+^, water, and acid–base balances, the renal primary defects or the extrarenal secondary perturbation affecting this segment have major consequences and some of them will be described in the following parts (Figs 5 and 6 and Table 1). As renin and aldosterone specifically target the CD, disorders affecting their secretion can lead to altered CD transports. These are extensively described elsewhere, as well as the effect of excessive cortisol production or glucocorticoid therapy (see reviews [27, 28]). The dysfunctions of the transport systems present in CD have been extensively studied these last decades, providing not only important data for the clinical management of patients but also allowing a better understanding of the renal physiology.

**Table 1:** Recapitulation of the primary and secondary disorders affecting the collecting duct

Disease	Main transport alteration	Clinical and direct biological consequences	Collateral consequences
Liddle syndrome	ENaC gain of function	Hypertension	Hypokalemia, metabolic alkalosis
Type 1 pseudohypoaldosteronism	MR inactivation ENaC loss of function	Salt wasting, hypotension, growth retardation	Hyperkalemia, metabolic acidosis
Apparent mineralocorticoid excess	Genetic 11βHSD2 loss of function	Hypertension	Hypokalemia, metabolic alkalosis
Congenital nephrogenic diabetes insipidus (NDI)	V2R loss of function AQP2 loss of function	Polyuria–polydipsia, hypernatremia	Sodium wasting?
Nephrogenic syndrome of inappropriate antidiuresis	V2R gain of function	Intracellular volume increase, hyponatremia	Extracellular volume increase?
Congenital distal renal tubular acidosis	H^+^‐ATPase loss of function	Metabolic acidosis	Hypokalemia, sodium wasting, water wasting, nephrocalcinosis, hypercalciuria
Lithium‐induced NDI	AQP2 expression and membrane addressing downregulation	Polyuria–polydipsia, hypernatremia	Sodium wasting
Nephrotic syndrome	ENaC and ASIC2A activation	Edema, sodium retention	Hyperkalemia
Glycyrrhizin intoxication	Acquired 11βHSD2 loss of function	Hypertension	Hypokalemia, metabolic alkalosis, water loss
Hyperaldosteronism (primary or secondary) Hypercorticism	MR activation by aldosterone or by excessive cortisol production or treatment by analogs	Hypertension	Hypokalemia, metabolic alkalosis, water loss
Trimethropin side effects Amiloride	ENaC inhibition	Hyperkalemia	Sodium wasting
Acquired distal renal tubular acidosis (Sjögren syndrome)	H^+^‐ATPase downregulation	Metabolic acidosis	Hypokalemia, sodium wasting, water wasting, nephrocalcinosis, hypercalciuria
Hypokalemia	Renal or extrarenal losses	Neuromuscular paralysis, cardiac arrythmia	NDI (water loss, polyuria–polydipsia), sodium wasting, metabolic alkalosis

## Primary perturbation of the CD

### Primary dysfunction of ion transport systems in the PC

*Liddle syndrome*. As mentioned above, the electrogenic Na^+^ reabsorption mediated by ENaC is a cornerstone of the renal ion transport system. ENaC consists of three subunits encoded by three different genes (*SCNN1A*, *B*, and *G*). The product of these genes are proteins with two transmembrane domains that assemble together to form a functional channel at the apical membrane of the cell. Mutations of these genes have been reported that either hyperactivate or inhibit the channel activity.

Liddle syndrome is an autosomal dominant disease characterized by early onset of hypertension and associated with hypokalemia. This syndrome is also known as pseudoaldosteronism or type 1 pseudohyperaldosteronism because it mimics primary hyperaldosteronism, but conversely to what would be expected, the plasma renin activity and aldosterone are very low, which indicates that the Na^+^ reabsorption in CD is constitutively activated. First described in 1963 [29], the autosomal dominant inheritance of this syndrome was established in 1994, when Botero‐Velez et al. investigated almost 50 parents of the index case [30]. In the initial case, a 16‐year‐old female exhibited resistant hypertension (180/110 mmHg) with profound hypokalemia (2.8 mM) and metabolic alkalosis with a low level of aldosterone that was modified neither by a low salt nor by a high K^+^ diet. Moreover, inhibition of the MR with spironolactone did not correct the symptoms whereas the use of triamterene, a blocker of the epithelial Na^+^ channel, did normalize blood pressure and plasma K^+^ value. Genetic analysis revealed that Liddle syndrome was due to mutations in ENaC subunits, mainly ENaCβ and γ (*SCNN1B* and *SCNN1G* genes), leading to the modifications (generally truncation) of a prolin‐rich sequence called PY motif, in the C‐terminal part of the protein [30, 31]. This PY motif is important for protein–protein interaction since it interacts with the WW domain carried by other proteins like the E3 ubiquitin ligase, Nedd4.2. This E3 binds to ENaC subunits and leads to its endocytosis and degradation [32] by the proteasome after ubiquitylation. This negative regulation of the channel activity is not possible with mutations in the PY motif. The overactivation of ENaC in Liddle syndrome is therefore due to a default of internalization and accumulation of an active channel that continuously reabsorbs Na^+^ [33]. A mouse model of Liddle syndrome was then developed by genetically mimicking a human mutation in the ENaCγ subunit, which led to the generation of a truncated protein in C‐terminus [34, 35]. This model exhibits an increased activity of ENaC both in the kidneys and colon but develops hypertension only when exposed to a high‐salt diet. Among the few tens (less than a hundred) of families investigated, the most common mutations are, indeed, in the PY motif. However, there are few reports of mutations that do not impede ENaC internalization and degradation but rather interfere with proteolytic cleavage and/or inhibition of the Na^+^‐dependent ENaC downregulation [36, 37].

*Pseudohypoaldosteronism type 1*. Conversely to Liddle syndrome, pseudohypoaldosteronism (PHA1) is characterized by mild to profound salt wasting and hyperkalemia, which may mimic the absence of aldosterone. However, the level of aldosterone and renin in these patients is elevated, indicating that the responsive tissues are rather resistant to this hormone. After the first observation of PHA1 in 1958 [38] and subsequent cases, it was established that this rare syndrome can be divided into two categories, the autosomal‐dominant PHA1, related to loss‐of‐function mutations of the MR (*NR3C2* gene) [39] and the recessive form related to mutations in ENaC, abrogating its activity [40]. The autosomal dominant form of PHA1 is also known as renal PHA1 since the loss of salt is restricted to the kidneys and is the most frequent one, with a prevalence of 1/80000 [41]. It is rather mild and spontaneous remission generally occurs at an adult age [42]. Mutations of the MR occur in all of the 10 exons of the *NR3C2* gene and lead to a variety of dysfunctions, from mRNA instability to protein degradation or loss of function such as alteration in the DNA‐binding capacity.

The systemic, autosomal recessive type of PHA1 is more severe and persistent all along the lifetime, affecting all organs/tissues expressing ENaC (kidneys, lungs, colon, salivary and sweet glands, etc.). The link between mutations identified in patients in the *SCNN1A* gene and the loss of function of ENaC has been described for a few of them exhibiting different characteristics. For instance, a missense mutation in the N‐terminus part of the ENaCβ subunit induces a strong decrease of ENaC activity after co‐expression with α and γ subunits in Xenopus oocytes by affecting the open probability of the channel and neither its single channel conductance nor its expression at the cell surface [43]. Another mutation in the ENaCα subunit (R508stop) gives rise to a truncated form with only the N terminal tail and the first transmembrane domain [44]. Surprisingly, after expression in Xenopus oocytes, what appeared to be a strong structural modification leads actually to a mild functional effect since this truncated form, combined with ENaCβ and γ subunits, has only slight differences with the wild‐type form. The single channel conductance was conserved but the density at the cell surface was lower, leading to a macroscopic loss of channel activity. It seems possible that the residual activity of ENaC observed in PHA1 was sufficient to avoid the dramatic phenotype observed in mice with a total absence of ENaC expression [45].

*The 11beta hydroxysteroid deshydrogenase type 2 (11βHSD2) loss of function: apparent mineralocorticoid excess syndrome*. The regulation of Na^+^ reabsorption and K^+^ secretion in the PC of the CD is under the control of aldosterone through the activation of the MR. However, this receptor is also sensitive, with a similar affinity, to glucocorticoids like cortisol (corticosterone in rodents). These glucocorticoid compounds are 1000 times more concentrated in plasma than aldosterone; therefore, the MR should be constantly activated if the PC did not express an enzyme, the 11βHSD2, that metabolizes cortisol into an MR inactive compound, cortisone. This crucial function impedes the constant activation of the MR system, hence massive reabsorption of Na^+^ and loss of K^+^ that would induce hypertension and hypokalemia. However, [46, 47] autosomal recessive mutations of 11βHSD2 have been reported, leading to childhood‐onset hypertension, hypokalemia, and alkalosis with low plasma renin and low plasma aldosterone levels (this last feature allows clinicians to differentiate these symptoms from hyperaldosteronism). Since the first case was reported in 1977 [48], around 40 mutations of 11βHSD2 have now been reported leading to severe or mild phenotypes depending on their impact on the activity of the enzyme [49]. Thus, for example, mutations affecting the NAD^+^ binding site lead to complete inactivity of the enzyme and correlate with a severe phenotype [50] whereas others only decrease the affinity [51] or the activity [52] of the 11βHSD2 and induce less severe forms of the disease. An elegant structure–function study using the elaboration of a 3D homology model of the 11βHSD2 allowed to predict the severity of novel mutations [47].

*Vasopressin receptor type 2 and AQP2 loss‐of‐function mutations: diabetes insipidus*. There are four entities (central, nephrogenic, gestational, and primary polydipsia) that lead to excretion of large amounts of hypoosmotic urine, a condition known as diabetes insipidus. These different forms of diabetes insipidus have been reviewed very recently [53]. Here, we will focus on the nephrogenic diabetes insipidus (NDI), which can be seen as a renal resistance to vasopressin (AVP), the antidiuretic hormone. This disease can either be inherited or acquired (mainly caused by lithium therapy, see below). Most (>90%) of the inherited NDI cases are related to loss‐of‐function mutations of the *AVPR2* gene encoding for the vasopressin receptor type 2 (V2R), expressed at the basolateral side of the PC in the CD. The remaining 10% are linked to mutations in the *AQP2* gene.

Since the *AVRP2* gene is carried by the X chromosome, male patients suffer a complete AVP resistance whereas females can be affected to various degrees. In PC of the CD, AVP triggers water retention through the activation of its V2 receptor by a signaling pathway involving stimulation of adenylyl cyclase, increase of cAMP (cyclic adenosyl monophosphate) production, activation of the protein kinase A, and phosphorylation of AQP2. This phosphorylation induces the exocytosis of AQP2 to the apical membrane of PC and allows water uptake by these cells. Water exits the PC at the basolateral side through AQP3/4. The mutations of V2R completely impede this signaling pathway and therefore strongly affect water reabsorption. There are around 300 disease‐causing mutations of the V2R, most of them in the transmembrane domains, that may either induce nonsense/missense (for about 60% of them) or frameshift, deletion/insertion, and so on (for a review, see [54]). These mutations affect the function of the receptor differently and have been grouped accordingly. Some mutations only impede the ligand binding properties (group I), others interfere with the cell surface expression of the receptor (group II), and finally, some affect the stability of the mRNA, leading to its degradation (group III). Interestingly, it seems possible to rescue the type II mutants, since, if they reach the plasma membrane, the receptors are functional [55, 56]. One strategy consists of applying cell permeant selective nonpeptide vasopressin receptor antagonist to stabilize the structure of the receptor, which probably allows it to pass the reticulum control quality system [56]. Other types of treatment have been proposed and tested in small groups of patients (for review, see [57])—strategies to increase intracellular cAMP or use of statins, acetazolamine, or metformin. However, all these approaches that directly target the affected signaling pathway have failed to prove a real benefit and suffer from a relatively low number of participants.

Loss‐of‐function mutations of AQP2 also lead to NDI and can have different modes of inheritance. The recessive mutations induce misfolding of the protein, impact the function of the water channel, and lead to its retention in the endoplasmic reticulum [58]. Interestingly, as in many cases of misfolded and ER‐retained mutants, the use of a chemical chaperone, like glycerol, can rescue the defect of targeting [59]. The dominant mutations result in functional but misrouted proteins to cellular compartments other than the plasma membrane. Since AQP2 is a heterotetramere, the presence of a mutated AQP2 in this complex is sufficient to mislocalize the nonmutated forms [60].

*V2R gain‐of‐function mutations: nephrogenic syndrome of inappropriate antidiuresis*. Nephrogenic syndrome of inappropriate antidiuresis (NSIAD) is characterized by the inability of the kidney to excrete free water and by the development of hyponatremia resembling an inappropriate excess production of AVP. However, in 2005, two independent cases were reported, exhibiting these symptoms but with a low/undetectable plasma AVP level [61]. Genetic investigations revealed that these two patients carried mutations on the *V2R* gene in a very conserved residue (Arg137) already known to induce NDI when mutated [62]. Heterologous expression of these mutants results in a constitutively active receptor and can trigger a vasopressin signaling pathway in the absence of the peptide hormone [63] and activate AQP2 through a PKA independent pathway [64]. An intuitive strategy for such a syndrome would be to provide a V2R antagonist such as tolvaptan, but this treatment is ineffective for NSIAD [65]. It seems rather more efficient to place the patients on fluid restriction and urea supplementation [66].

### Primary dysfunction of the ion transport system in IC

*Distal renal tubular acidosis*. The inability of the kidney to appropriately excrete protons leads to metabolic acidosis with a normal plasma anion gap characterized by a low plasma bicarbonate and pH. This is a rare acid–base disorder (one in 100,000) having other consequences such as hypokalemia and the development of nephrocalcinosis or urolithiasis [67] since a high urine pH favors the deposition of calcium phosphate crystals. The defects in the kidney could be located either in the proximal or the distal segment of the nephron and affect either the secretion of proton in the lumen or the reabsorption of bicarbonate. As mentioned above, in the CD, these functions are carried by the ICA. Mutations of ion transporters or regulators that govern H^+^ and bicarbonate transport in this cell type have been reported to be at the origin of the inherited forms of metabolic acidosis (Fig. 6). The mutations of five genes have been described in this setting. Two genes, *ATP6V1B1* [68] and *ATP6V0A4* [69], encode proteins constituting the multimeric complex forming H^+^‐ATPase; one encodes the AE1 transporter (Slc4a1) [70] mediating the reabsorption of bicarbonate and two others encode a transcriptional factor (the forkhead transcription factor FOXI1) [71] involved in the cell specificity of the ICA and a regulator of protein trafficking (the tryptophan–aspartate repeat domain 72, WDR72) [72]. The mutations in the H^+^‐ATPase subunits are the most common ones causing distal renal tubular acidosis (dRTA) and these mutations may also lead to deafness [68, 73]. These mutated subunits lead to a loss of function of the H^+^‐ATPase. Mice invalidated for *ATP6V1B1* [74] do not exhibit a strong phenotype under normal conditions, possibly due to a compensation by the expression of *ATP6V1B2*, a subunit normally found in vacuoles but observed at the apical membrane of ICA in null animals [74, 75]. However, B1 KO (knock‐out) mice exhibit a defect in response to an acid load. *ATP6V0A4* KO mice exhibit a strong dRTA phenotype with metabolic acidosis, hypokalemia, and nephrocalcinosis [76], and also display hearing impairment [77]. Regarding AE1 pathogenic mutations, in vitro studies using a heterologous expression system such as Xenopus oocytes or Madin‐Darby Canine Kidney (MDCK) cells have revealed that dominant R589H or S613F mutants were sequestered into intracellular compartments [70, 78]. Interestingly, when expressed in nonpolarized cell systems such as oocytes or erythrocytes, some mutants (G609R, M909T) did not show any difference with WT AE1 [70]; however, their study into polarized cells showed that they were mistargeted to the apical membrane [79, 80]. The lack of function of AE1 was proved to be responsible for dRTA since the AE1‐null mice exhibited most of the human phenotype associated with AE1 mutations [81]. As mentioned above, some mutated genes are not directly related to ion transporters. This is the case with *FoxI1* encoding a transcription factor that is responsible for the fate of CD cells. Indeed, as mentioned earlier, CD is composed of different cell types expressing specific proteins such as AQP2 for the PC and AE1 or type II carbonic anhydrase in IC. In the absence of FoxI1, there is only one type of CD cells that expresses markers of both PC and IC, indicating that FoxI1 is required for proper patterning of this renal segment [82]. Moreover, the mice lacking FoxI1 also display metabolic acidosis and are not able to handle acute or chronic acid load properly [82], which may be related to the fact that this transcription factor also regulates expression of different subunits of the H^+^‐ATPase [83]. Regarding *WDR72*, this gene encodes a protein mainly known for its role in the amelogenesis whose dysfunction leads to amelogenesis imperfecta [84] but also to metabolic acidosis [80]. The role of WDR72 in kidneys is still not known and its expression has not been clearly established but an RNA‐seq database analysis showed that expression of WDR72 is present in all three cell types of the CDs. There is still no mechanistic explanation for rely mutations in *WDR72* genes and their renal consequences.

Conversely to other causes of metabolic acidosis, hypokalemia is a central feature of type I RTA and can be profound. Its mechanism is not completely understood. Enhanced potassium excretion might be the result of an increase in lumen negative voltage in the CD due to a lack of H^+^ secretion through the H^+^‐ATPase [85]. The coordination between IC and PC appears, however, to be a major regulator of sodium, potassium, and water transport. This view is strengthened by, on the one hand, clinical evidence of sodium loss in patients with dRTA [86] and on the other hand by the study by Gueutin et al. [87] showing, in a model of dRTA, sodium, potassium, and water loss by PC through a paracrine PGE2 signaling cascade leading to the suppression of both ENaC and pendrin expression.

## Secondary perturbation of the CD

The CD is also a target of the environment (pharmacological treatment, immune system, diet, etc.), which may cause perturbations of its transport systems, leading to dysregulation of electrolyte and water balances. In the following parts, we will describe some examples of such an interaction and their pathological consequences.

### Secondary dysfunction of the ion and water transport system in PC

*Lithium intoxication*. Lithium is the cornerstone treatment of bipolar disorder, characterized by potentially life‐threatening manic and depressive episodes. One of the main adverse effects of this treatment is NDI leading to polyuria and polydipsia, occurring in 20%–70% of patients depending on the population and the definition of NDI [88]. As a monovalent cation, lithium follows sodium transport along the kidney tubule and enters the PC of the CD via ENaC [89]. Interestingly, by comparing the effects of lithium among 29 inbred strains of mice, De Groot et al. [90] have recently shown that the polymorphism of the *ACER2* gene was a risk factor for aggravated NDI, probably through an overexpression of ENaC. Studies using murine models clearly show that very early after lithium initiation, AQP2 expression and apical membrane addressing decrease [91], which is consistent with the observation of a decrease of AQP2 in urine from patients treated with lithium [92]. The downregulation of AQP2 is not completely understood but implicates lithium‐induced GSK3 inhibition [93], which partly explains its thymoregulatory effect in neuronal cells. Increase in PGE2 production has also been reported as an additional mechanism explaining polyuria [93]. Our recent study identified the daily dose as the main determinant of vasopressin resistance in lithium‐treated patients, hence suggesting that targeting the minimally effective dose might be of interest in this setting [94].

In addition to water, sodium transport is probably also altered during lithium therapy. Deen et al. [58] and Nielsen et al. reported a decrease in ENaC expression in lithium‐treated mice, leading the authors, consistent with other reports [95], to provide mice with salt blocks [96]. Noteworthy, the increase in vasopressin due to vasopressin resistance in PC might confer V1aR‐mediated sodium and water diuresis, further aggravating the phenotype [97].

Interestingly, an interplay between PC and IC has been identified during lithium therapy, as; a proliferative effect on the CD leads to a marked increase in the ICA–PC ratio [98, 99]. This huge increase of ICA could result in perturbation of the acid–base balance under lithium treatment; however, data regarding tubular acidification are conflicting, with no overt change in renal ammoniogenesis or proton excretion by IC [100].

*Nephrotic syndrome*. Nephrotic syndrome is a congenital or acquired condition characterized by massive proteinuria and hypoalbuminemia. It includes two histological variants—minimal change nephrotic syndrome and focal and segmental glomerulosclerosis. While congenital nephrotic syndrome has a genetic basis, the pathogenesis of acquired nephrotic syndrome is still under investigation, with the potential implication of immune cell dysregulation, podocyte dysfunction, and/or the release of a soluble factor resulting in the disruption of the glomerular filtration barrier [101]. Nephrotic syndrome is associated with a state of extracellular volume increase, in particular in the interstitial space, resulting in important edema associated with massive proteinuria and hypoalbuminemia. The classically described mechanisms explaining salt retention in this context involve a decrease in oncotic pressure due to hypoalbuminemia being the putative cause of this edematous state. The resulting decrease in plasma volume would lead to RAAS activation and sodium retention through activation of ENaC. This “underfill theory” has been weakened by clinical observations suggesting, on the contrary, an “overfill” state where there is no hypovolemia, and where sodium reabsorption is intrinsically stimulated by nephrotic syndrome [102]. This “overfill theory” is currently the most prominent as aldosterone levels are only transiently elevated after the onset of nephrotic syndrome, and since the MR antagonist failed to inhibit Na^+^ retention [103]. Moreover, it was shown in puromycin aminonucleoside (PAN)–treated rats, a well characterized model of nephrotic syndrome, that adrenalectomy and aldosterone clamp did not impede Na^+^ retention and ascite formation, indicating that these effects are independent of mineralocorticoid [104]. Moreover, increased sodium reabsorption can be induced in isolated kidneys treated with PAN, hence independently of perturbations in the RAAS system [105].

Two transporters have been identified in the pathophysiology of increased sodium reabsorption in the kidney. When aldosterone is high, ENaC subunits and their cleavage increasing ENaC activity has been identified as one potential mechanism explaining the increase in sodium retention. Filtered proteases during glomerular proteinuria might thus explain ENaC activation, along with aldosterone increase in a subset of patients [106]. More recently, in the context of a normal plasma aldosterone level, another potential mediator of sodium retention has been identified, represented by a truncated variant of acid‐sensing ion channel 2b (ASIC2b) that induces acid‐stimulated sodium currents when coexpressed with ASIC2a [107]. This channel is sustainably activated by albumin endocytosis in the CD of nephrotic rats and is specifically expressed in kidney biopsies from nephrotic patients.

Activation of electrogenic Na^+^ reabsorption mechanisms (ENaC or Asic2a) should stimulate ROMK‐dependent potassium excretion in the CD. Several lines of evidence suggest quite the contrary. Nephrotic rats display ROMK inhibition and inability to excrete a dietary load of potassium [108]. In the same line, nephrotic patients have elevated plasma potassium levels independent of GFR decline before corticosteroid initiation [109].

*Alteration of the Na^+^ reabsorption process by exogenous compounds, for example of the glycyrrhizic acid and trimethoprim (Bactrim^®^)*. As mentioned above, the main actors of Na^+^ reabsorption in the PC are the channel ENaC, the MR, the nuclear receptor of aldosterone, and the 11βHSD2 enzyme that converts cortisol into cortisone and protects the MR from inappropriate activation by glucocorticoids. The ingestion of licorice, containing glycyrrhizic acid, as confectionary or medication has been shown to induce hypertension and hypokalemia. At first, it was thought that glycyrrhizic acid or its metabolite glycyrrhenitic acid directly binded and activated MR but it was demonstrated that the effect of these compounds was not observed in the absence of adrenal glands [110]. Further studies showed that glycyrrhenitic acid inhibited the function of the 11βHSD2 enzyme, allowing activation of the MR by cortisol [111].

Trimethoprim–sulfamethoxazole is a wide‐spectrum antibiotic that is known to induce hyperkalemia in treated patients [112, 113, 114]. Using animal models, it was demonstrated that treatment with trimethoprim inhibited Na^+^ transport in frog skin [115] and, when injected in rats, increased natriuresis and decreased K^+^ secretion [112]. All these data converged with an inhibitory action on the ENaC channel, which was confirmed by Muto et al. using microperfusion of rabbit CDs [116], with a similar effect to amiloride, which is a specific ENaC blocker used as a potassium‐sparing diuretic treatment.

### Secondary dysfunction of the ion transport system in IC

*Autoimmune tubular acidosis: Sjögren syndrome*. dRTA is a well‐known complication of Sjögren syndrome. It can be complete or incomplete, characterized by normal plasma anion gap metabolic acidosis and alkaline urinary pH at various degrees. It has been reported in 3%–25% of patients with Sjögren syndrome, but up to 73% of patients with confirmed renal involvement [117, 118], and is correlated with a higher prevalence of circulating auto‐antibodies. Immunohistochemical analysis of kidney biopsies from patients has consistently shown a decrease in H^+^‐ATPase expression in IC within the CDs [119]. As a proof of concept, Devuyst et al. [120] elegantly showed that purified IgG from the serum of a patient with Sjögren syndrome and dRTA detected IC of human control kidneys, suggesting a specific reactivity of at least a part of these IgG. Anticarbonic anhydrase II auto‐antibodies have also been related to dRTA in a subset of Sjögren patients [121], but they do not seem specific and other targets are possible like carbonic anhydrase VI or XIII [122]. In the same line as in the primary dRTA described earlier, sodium, potassium, and water transport are altered along with the main presentation (see above).

### Secondary dysfunction of the ion transport system of both PC and IC

*Hypokalemia*. Hypokalemia is associated with various electrolyte and water transport adaptations and can be due to extrarenal causes such as potassium depletion during anorexia, potassium losses during acute or chronic diarrhea, or renal potassium losses [123]. The latter are exclusively related to distal nephron potassium wasting from various mechanisms, including aldosterone‐mediated sodium reabsorption activation.

When hypokalemia is not the result of an excess of mineralocorticoid leading to an inappropriate renal K^+^ secretion, it leads to a decrease of aldosterone inducing a downregulation of ENaC expression and electrogenic Na^+^ reabsorption. Reabsorption of potassium through the IC H,K‐ATPase type 2 is stimulated by a progesterone‐dependent mechanism [20], hence promoting urinary acidification. Moreover, this ability to reabsorb K^+^ is improved by the increased number of ICA due to proliferation [124] and/or transdifferentiation of PC into ICA [125]. Finally, polyuria is commonly reported during potassium restriction and hypokalemia in murine models as well as in human case series [126]. This is related to a state of NDI, with a decrease in AQP2 expression and vasopressin resistance [127] independent of sodium reabsorption, that may be related to autophagic degradation of AQP2 [128]. The correction of potassium depletion is accompanied by the resolution of NDI.

*Water intake*. Water intake determines urine dilution in nonextreme environments. In situations of poor water intake, urine concentrates through the action of vasopressin, which is released by the posterior pituitary following a rise in serum osmolality. Consequently, four variables have been studied to determine the association between higher urine concentration and adverse clinical outcomes: water intake, urinary output, urine osmolality, and vasopressin levels (for review, see [129]). The latter is commonly replaced by its surrogate marker, copeptin, which is cosecreted with vasopressin in equimolar amounts [130]. Noteworthy, consumption of water has become an issue, not only in the drinking water supply of desertic countries or in developing countries, but also in developed countries where intake of sugar‐sweetened beverages increases dramatically, mainly among the youngest [131, 132, 133]. Recurrent or acute water dehydration is a risk factor for acute and chronic kidney injury, as suggested by the emerging epidemics of chronic kidney disease (CKD) in agricultural communities of Central America [134]. The putative identified mechanisms include the effects of hyperuricemia, of the activation of the aldose reductase–fructokinase pathway, and of the elevation of vasopressin levels [135]. High urine osmolality and low water intake are well‐known risk factors of urolithiasis, but are also associated with other outcomes such as cardiovascular mortality, CKD, and end‐stage kidney disease (ESKD) (for review, see [136]). Indeed, increased vasopressin release is necessary in order to excrete high amounts of osmoles in a minimal volume of water. Through its ligation via V1a and V2 receptors, vasopressin exerts adverse effects on the kidney, as shown in various experimental models [137]. In humans, previous studies have reported an association between adverse outcomes and elevated copeptin levels [138, 139]. Of note, V1aR‐ and V2R‐mediated signalization have opposite effects on urinary output, explaining the differential effect of water intake and specific V2R antagonism [97]. Tolvaptan, a selective V2R antagonist, has a protective effect on the progression of cysts in polycystic kidney disease [140]. The medicinal use of water in order to suppress the action of vasopressin is a matter of debate [141], although it has been demonstrated, in interventional studies, that increasing water intake reduced not only copeptin but also glucagon levels, suggesting an effect on glucose metabolism [142]. A recent study, however, showed that a higher water intake during PKD did not result in slower cyst progression or lowering of copeptin values [143]. The major issue in the interpretation of clinical trials and epidemiological data in polcystic kidney disease (PKD) and CKD patients is that urine concentrating ability progressively decreases in the progression of these diseases due to kidney medullary and microvascular dysfunction [144, 145]. This altered urine concentration leads to hyposthenuria, thus resulting in higher water intake. Clinical data should thus be interpreted with caution and in the light of potential reverse causality. As a matter of fact, CKD‐ or PKD‐induced hyposthenuria raises vasopressin levels as a result of kidney resistance to its action, which in turns might cause deleterious effects [145].

## Therapeutic section

Advances in the understanding of these transport alterations have helped inform optimal management of patients. This is well illustrated by the specific blocking of ENaC by amiloride, for instance, in the treatment of Liddle syndrome [146]—due to primary hyperactivation of ENaC—or lithium‐induced NDI—due to lithium entry in the PC via ENaC [92]. Conversely, in mutations of 11βHSD2, management is based on MR antagonism, as excessive cortisol activates MR in PC [147]. Experimental data have also opened potential therapeutic avenues, for example, in nephrotic syndrome. As stated above, salt retention is not exclusively driven by aldosterone‐mediated MR activation, or downstream by direct activation of ENaC. As a matter of fact, MR antagonists and amiloride have shown inconsistent efficacy in the treatment of edema during nephrotic syndrome [148]. In this view, the identification of ASIC2a as a key contributor paves the way to new therapeutic targets. Finally, blocking vasopressin signaling has not only proven to be efficient in reducing cyst growth in PKD, but might also be a promising strategy in the prevention of renal function decline in chronic kidney disease [136].

Deciphering the lines of communication between PC and IC has helped unravel the common association of water, electrolyte, and acid–base imbalances (summarized in Table 1). These findings also contribute to improvement in therapeutic strategies in patients with these disorders. As such, salt management (restriction or supplementation) is often mandatory to treat potassium defects and hypokalemia should be treated to restore urine concentrating ability. Although not fully established, previous reports have suggested that alkalinization per se should reverse hypokalemia during dRTA, as acidosis increases distal delivery of Na^+^, further aggravating hypokalemia [149]. Taken together, the understanding of the pathophysiological processes resulting in water, electrolyte, or acid–base disorders should be considered as a prerequisite to their management and treatment.

## Conclusion

Alterations of ion transport systems in PC (Fig. 5) or IC (Fig. 6) of the CD are at the origin of many different diseases having global repercussions on the organism by inducing modifications of electrolyte balances (Table 1). These last decades, effort has mainly been focused on the identifications of the dysfunctional components of the ion transport system, with a lot of success, as reviewed above. To better understand the mechanisms of theses tubulopathies and the diversity of symptoms that are, in many circumstances, not related to the genotype, we believe that further investigations should now be orientated toward the analysis of the consequences of these primary or secondary perturbations. To understand how these dysfunctions of the ion transport systems impact the cell physiology, its metabolism, and its proliferative rates, how they induce ER or oxidative stresses and how they evolve in time may be an interesting field of research to better characterize and manage these tubulopathies.

## Conflict of interest

The authors report no conflict of interest in relation to this publication.

## Author contributions

Nahid Tabibzadeh: Writing – original draft; Writing – review and editing. Gilles Crambert: Writing – original draft; Writing – review and editing.
